# Antioxidant Therapy: Is it your Gateway to Improved Cardiovascular Health?

**DOI:** 10.4172/2153-2435.1000323

**Published:** 2014-12-29

**Authors:** M Ruhul Abid, Frank W Sellke

**Affiliations:** 1 Cardiovascular Research Center, Department of Surgery, Rhode Island Hospital, Brown University Warren Alpert Medical School, Providence, RI, USA

**Keywords:** Antioxidants, Coronary artery disease, Oxidative stress, Endothelial health, Oxidative response

## Abstract

General use and popularity of over-the-counter supplemental antioxidants have rapidly spread all over the world and are believed to promote cardiovascular health and wellbeing. However, there is a paucity of information and lack of proof that physiological and above-physiological levels of oxidants do harm at the cellular and organismal levels. Instead, several reports demonstrated that reduction in Reactive Oxygen Species (ROS) did not improve vascular function. Interestingly, recent studies show that increased ROS levels play protective role in vascular endothelium and may improve coronary endothelial function. In the current review, we introduce the concept that increased ROS levels, often seen in association with cardiovascular disease, probably is an endothelial-way or ‘oxidative response’ to cope with vascular pathology.

## Introduction

Vascular health depends on both structural and functional well-being of the blood vessels. Pathological changes can take place at the level of vascular endothelium, vascular smooth muscle cells (VSMC) and connective tissue surrounding the blood vessels [1–4]. Decreased bioavailability of nitric oxide (NO), resulted from decreased synthesis of NO, reduced activation of endothelial nitric oxide synthase (eNOS) or increased quenching of NO by reactive oxygen species (ROS) [5], is believed to be one of the major determinants of microvascular endothelial dysfunction in aging, hypertension, diabetes, hyperlipidemia, and smoking [6–9]. Other pathological changes that follow endothelial dysfunction may include increase in microvascular tone, neointimal thickening, myogenic hypertrophic remodeling of resistance arterioles and small arteries [2,10].

## Oxidants and vascular health

**Figure 1:**
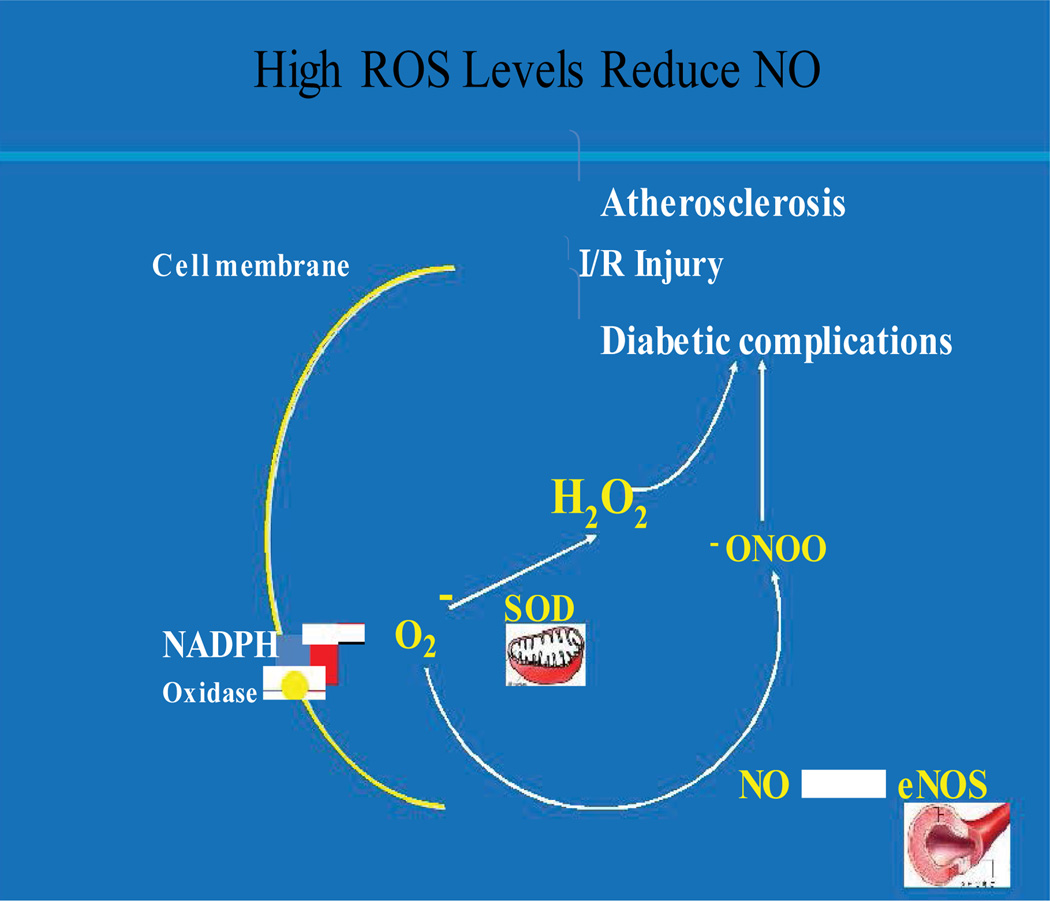
Sources of Endothelial ROS and NO It also demonstrates how increased ROS may tip the balance of NO *vs.* ONOO in vascular pathological conditions.

The notion that increased levels of reactive oxygen species (ROS) are detrimental to cardiovascular health has come into being for several reasons (Figure 1). Higher levels of ROS are often observed with microvascular pathology in Cardiovascular Diseases (CVD) including Coronary Artery Disease (CAD) and Ischemic Heart Disease (IHD) [11–15]. At first, these observations helped establish the paradigms that reduction in ROS levels in the vessel walls should improve cardiovascular functions [16]. However, several clinical trials using antioxidants, e.g. Alpha-Tocopherol Beta-Carotene (ATBC), Heart Outcomes Prevention Evaluation (HOPE) [17–22], have produced negative results in reducing primary endpoints of cardiovascular mortality and morbidity [17,23–26]. Other studies using animal models and/or antioxidants in Endothelial Cells (ECs) demonstrated that reduction in ROS levels failed to improve vascular functions [27–29]. Recent reports from several groups showed that ROS reduction resulted in the disruption of signal transduction leading to reduced Nitric Oxide (NO) generation in vascular endothelium [30–32] and decreased vasodilatation [30,31]. Surprisingly, a recent report demonstrated that increase in EC-specific ROS induces AMPK-eNOS-mediated endothelium-dependent coronary vasodilatation, and AMPK-mTOR-mediated protective autophagy [33].

## The redox paradox

So, why there is still a notion that ROS are harmful for cardiovascular health? There is no simple answer to this question. One reason would be that (i) ROS are found at increased levels in pathological condition involving cardiovascular system including CAD, myocardial ischemia, and myocardial infarct; (ii) another may be that our initial understanding of ROS is associated with their bactericidal effects in phagocytes, and (iii) the fact that there are many in vitro studies using ROS-inducing chemical agents that demonstrated apoptosis and other damages in vascular cells including ECs and VSMCs. If we look carefully and systematically, we will find that the first ‘reason’ is simple association, the second one does not take cell type and phenotypic differences into consideration, and the last one can simply be nonspecific effects of the so-called ROS-inducing chemicals that may have several other ‘indirect’ effects on vascular cell signal transduction, cell cycle and/or metabolism. It is thus critical to examine the sources and functions of vascular ROS. We will mostly focus on EC-specific ROS in the current review. There are several sources for intracellular ROS in EC including NADPH oxidases, mitochondria, cytochrome P450 and xanthine oxidase. The multi-subunit NADPH oxidase, which contains membrane-bound gp91phox (Nox2) and p22phox subunits, and cytosolic p47phox, p67phox and Rac1, is a major source of endothelial ROS [34–36]. NADPH oxidase is present in different subcellular compartments in ECs including cell and perinuclear membrane, and endoplasmic reticulum (ER) [36,37]. Several other NADPH oxidases e.g. nox4, nox1, nox5 are also present in ECs [38–42].

**Figure 2:**
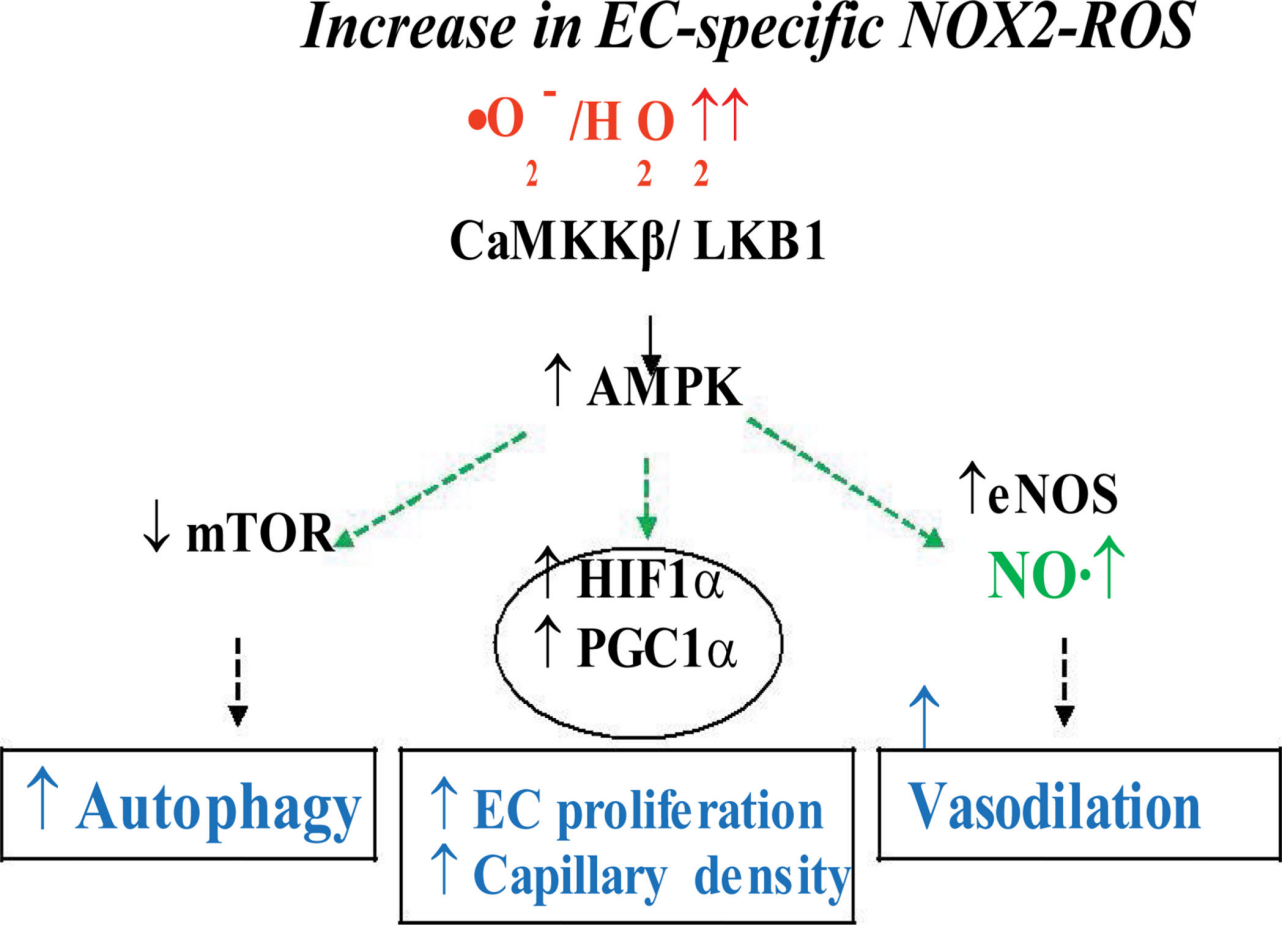
Model for EC-specific ‘Oxidative Response’ to improve endothelial function NADPH oxidase-derived ROS activates CaMKKβ-AMPK, which in turn, activates eNOS to induce NO-mediated vasodilatation and inhibits mTOR resulting in protective autophagy in vascular endothelium.

Recent work from others and our labs has shown a critical role for NADPH oxidase-derived ROS in the activation of downstream eNOS to synthesize NO [28,42–47]. Taken together, above findings suggest that NADPH oxidase-derived ROS play an important role in survival, health and growth of vascular ECs. We are yet to understand the precise mechanisms involving ROS-mediated signal transduction in ECs. Several reports showed that oxidants play crucial roles by activating signaling intermediates including PI3K-Akt-eNOS, PLCγ1, PKC and ERK1/2 in ECs [27,43,48]. Recently, pro-survival kinase AMPK that becomes activated during cellular stress including starvation and reduction in AMP/ATP ratio, has been shown to be regulated by ROS produced during hypoxia and fluid shear stress in ECs [49–51]. AMPK is involved in regulating a number of signaling intermediates and transcription factors including FOXO1, HIF-1α and PGC-1α [52–58], resulting in increased EC survival and proliferation (Figure 2). The protective role of AMPK is carried out through its regulatory role in autophagy, a process crucial for cell survival [51,59–62]. Autophagy helps recycle and re-utilize damaged macromolecules and organelles using lysosomal degradation pathway [59,63]. In a recent study from our lab using increase in EC-specific endogenous ROS in adult animals (a novel binary Tet-ON/OFF transgenic mouse) that induces 1.8 ± 0.42-fold increase in Nox2/gp91phox (NADPH oxidase 2)-derived ROS, we demonstrated that EC-ROS induced AMPK-eNOS-mediated endothelium-dependent coronary vasodilatation and AMPK-mTOR-mediated protective autophagy in EC [33]. However, several studies were performed using cultured ECs in vitro, global knockdown animal models of NADPH oxidase (p47phox−/− or gp91phox−/−), or constitutive overexpression of NADPH oxidases (e.g. Nox4) in ECs [28,35,43,44,64–66] that resulted in a wide variety of conclusions ranging from essential to harmful roles for ROS. These approaches, although yielding important information, may have precluded precise determination of endothelial contribution for redox-sensitive modulation of vascular functions. In brief, a reductionist view or an all-or-none theory may not apply to the roles for ROS in vascular function. One has to take the source, subcellular localization, intensity and temporal state of the redox molecule into consideration while studying the roles/effects of ROS in/on vascular function.

## Oxidative stress or oxidative compensation?

One may ask why does the prevailing dogma still support a negative role for ROS? To address this question, we need to go back to the origin of the oxidative stress ‘theory’ in cardiovascular system. We must remember that the notion that increased levels ROS are detrimental to cardiovascular health has come into being for ROS’ association with several different cardiovascular pathology including CAD, myocardial ischemia-reperfusion, and myocardial infarct. We know that the most potent way myocardium employs to defend itself from ischemic insults is by preserving the existing capillary endothelial cells (EC) and/or by inducing growth of coronary blood vessels in the ischemic area [67]. Once the ischemic insult has occurred, survival of the affected cardiac tissue depends on the speed with which coronary vessels can increase blood flow through alternate means, namely vasodilatation, increase in vessel density, and/or preservation of the existing microvessels. Given the recent findings by others and our lab (as mentioned above), we propose a novel concept that vascular endothelium addresses this critical issue by attempting to ‘pre-condition’ the coronary vessels for better vasodilatory and angiogenic response by increasing oxidant levels in the vascular cells. This concept, a ‘compensatory’ role for ROS, is in contrast to the prevailing dogma and suggests that redox positively regulates endothelial signaling and selective vascular functions [30,31,68–72] in health and disease. This novel ‘oxidative compensation’ concept also derives its support from the findings that NADPH oxidase-derived ROS, a major source of EC-ROS, have also been shown to play crucial role in vascular endothelial growth factor (VEGF) signaling and coronary vascular functions [30,31,70]. As a molecular mechanism, it was shown that c-Src activation and its interaction with VEGFR-2 were dependent on the redox-mediated thiol oxidation of c-Src (sulphenic acid modification) in human coronary artery ECs [27]. Recent findings that increase in endogenous EC-ROS enhance protective autophagy by activating AMPK further supports a pre -conditioning or compensatory role for ROS to lead ECs towards a pro-survival mode (Figure 2). AMPK is also known to be activated in a state of calorie restriction. Caloric restriction or nutrition deprivation slows down energy-consuming processes and induces autophagy to provide amino acids for the synthesis of essential proteins, which in turn improves cell survival and inhibits apoptosis under stressful conditions including high oxidant state in many cell types. Autophagy is essential for cellular survival, homeostasis, differentiation, and tissue remodeling in pathophysiological condition. Depending on the pathophysiological settings, autophagy may play a protective role or contribute to cell damage. Thus, our recent findings demonstrating increased ROS levels elicit a ‘caloric restriction’-like response (AMPK-mediated mTOR inhibition and induction of autophagy) in endothelium may also have important clinical implication [33] and may further support the notion that ROS play a compensatory role during vascular insult. Activation of AMPK and induction of autophagy may well be a mechanism by which endothelium copes with higher redox state by slowing down endothelial metabolism and/or by recycling the oxidant-damaged cellular organelles. This compensatory mechanism may more appropriately be termed as ‘oxidative response’ of vascular endothelium. Future studies are required to address the tissue- and vascular bed-specific response to oxidants, and the outcomes of these studies are likely to bring a major shift in our attitude towards the high ROS levels that are found in many microvascular diseases and may generate enthusiasm to examine whether high oxidant level in the microvascular wall in IHD/CAD and other microvascular pathophysiology is an undesirable by-product (resulting in oxidative stress) or a homeostatic response (oxidative response) to an inflammatory environment. The delicate balance between the beneficial (oxidative response) and detrimental (oxidative stress) roles of ROS may be compartmentalized within the cell (subcellular localization). In addition, different tissues and vascular beds may respond to oxidants differently depending on the types of oxidants (O_2_, H_2_O_2_, HO_2_, ONOO), their subcellular source/localization, intensity and duration of the exposure. Outcomes of the studies addressing these critical issues will help guide investigators whether or not to interfere with redox levels (e.g. by antioxidants) in coronary and other microvascular disease, which if used inadvertently, may paradoxically affect redox-dependent signaling and may thus predispose patients to further ischemia. In conclusion, one should not consider increased ROS level as an “all or none” phenomenon or as oxidative stress. Each condition with increased oxidant levels should be assessed independently before initiating any antioxidant therapy, because interference with oxidative response may do more harm than good.
